# The influence of AI on surgical and ablative treatments for colorectal liver metastases: a review of the current literature

**DOI:** 10.1186/s13244-025-02072-9

**Published:** 2025-11-12

**Authors:** Ariadne L. van der Velden, Robrecht R. M. M. Knapen, Hossein Rahmani, Leroy Volmer, Sorina R. Simon, Coosje A. M. Verhagen, Mark C. Burgmans, Andre L.A.J. Dekker, Joachim E. Wildberger, Ronald M. van Dam, Ralph Brecheisen, Christiaan van der Leij

**Affiliations:** 1 Department of Radiology and Nuclear Medicine, Maastricht University Medical Center, Maastricht, The Netherlands; 2 GROW, Research Institute for Oncology and Reproduction, Maastricht University, Maastricht, The Netherlands; 3 CARIM, Cardiovascular Research Institute Maastricht, Maastricht University, Maastricht, The Netherlands; 4 Department of Radiation Oncology (Maastro), Maastricht, The Netherlands; 5 Department of Radiology, Leiden University Medical Center, Leiden, The Netherlands; 6 Department of Surgery, Maastricht University Medical Center, Maastricht, The Netherlands; 7 NUTRIM School of Nutrition and Translational Research in Metabolism, Maastricht University, Maastricht, The Netherlands

**Keywords:** Colorectal liver metastases, Surgery, Thermal liver ablation, Artificial intelligence

## Abstract

**Objectives:**

Artificial intelligence (AI) has gained increasing interest in supporting clinicians in patient selection, treatment planning, and prognostics. However, the current application of AI in the treatment of colorectal liver metastases (CRLM) is not clearly established and validated. This scoping review assesses the current impact of AI techniques on local therapeutic strategies for CRLM, exploring its benefits, challenges, and future directions.

**Materials and methods:**

A comprehensive literature search in PubMed, EMBASE, Web of Science, and SCOPUS was conducted for patients with CRLM undergoing surgery or thermal ablation, along with descriptions of AI tools. Eligible studies were cohort studies or clinical trials. Data extraction focused on treatment strategies, AI techniques, clinical and radiomics parameters, and outcomes.

**Results:**

Out of 1464 articles, thirteen met the inclusion criteria. Eight articles regarded thermal liver ablation, and five surgical resection for CRLM. Most studies used traditional machine learning methods, such as support vector machines and random forests, combined with radiomics for predictive model building. While most studies demonstrated high performance, they frequently involved small sample sizes, and machine learning techniques often lacked robustness.

**Conclusions:**

AI shows promising results in improving local treatment strategies for CRLM, but further advancements are required for AI decision support tools. Future research should focus on large multicentre studies to validate AI-driven personalised colorectal liver metastases treatment strategies.

**Critical relevance statement:**

This review evaluates the use of AI in colorectal liver metastases treatment for outcome prediction and treatment evaluation. Included studies used AI for segmentation and predictive modelling and were often limited by small sample-sized single-centre studies, necessitating multicentre studies.

**Key Points:**

- The position of AI in local colorectal liver metastases treatments is not yet established.
- AI algorithms are mostly used for predictive model building and image registration.
- Studies often lack validity, limiting generalisability and implementation of AI support tools.

**Graphical Abstract:**

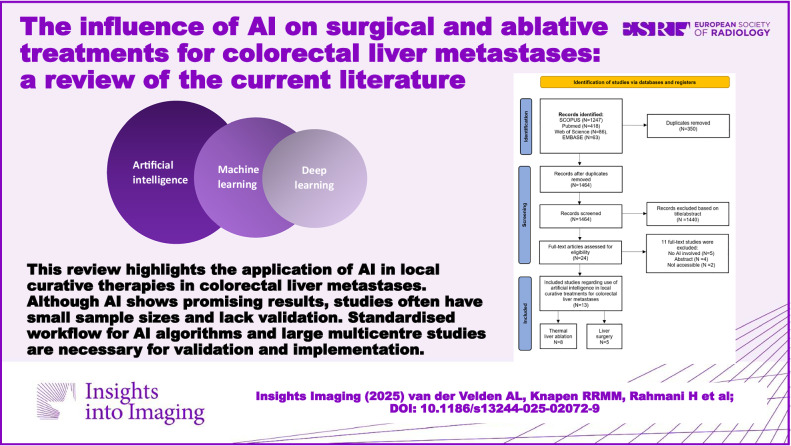

## Introduction

Colorectal cancer is the third most commonly diagnosed cancer worldwide [1, 2]. The liver is the most common site of metastatic disease (25–50%) [3–5]. For patients with colorectal liver metastases (CRLM), surgical resection is still considered the current standard for curative treatment [6, 7].

In recent years, alternative minimal invasive curative treatments such as radiofrequency ablation (RFA) and microwave ablation (MWA) have emerged. These thermal ablation (TA) techniques have been proven effective in terms of (local) technical and clinical success and improvement of overall survival, especially when applied to liver tumours of ≤ 3 cm [6]. Local tumour progression (LTP) remains a challenge [8]. Predicting LTP after resection or TA for CRLM could therefore be crucial in following treatment decisions [9]. Despite advancements in therapeutic options for CRLM, achieving optimal clinical outcomes remains challenging.

The management of CRLM requires a multidisciplinary approach [10, 11]. A study shows that, while clinicians exhibit high certainty (84%) regarding the predefined treatment strategy, alterations in the treatment strategy still occur in 36% of the cases, with 72% of these alterations being major, resulting in a change of the initial treatment after multidisciplinary meetings [12]. This may be attributed to the growing emphasis on patient preferences, resulting in more personalised treatment strategies. Developing a precision-medicine personalised approach to optimise survival and quality of life for patients can be challenging. One way to overcome these challenges is with the support of Artificial Intelligence (AI), more specifically, machine learning (ML). In recent years, the implementation of AI has gained increased interest in healthcare for its potential to improve diagnostics, patient selection, treatment planning, and clinical outcomes, as well as to support clinical decision-making.

AI encompasses technologies and applications that can simulate and mimic human intelligence, and is divided into subfields such as ML and deep learning [13, 14]. ML includes algorithms that learn from data analysis, which may be used for the development of prediction or decision models [13]. Deep learning is a subdivision of ML that utilises multi-layered algorithms that can simulate the complex decision-making ability of the human brain, without the necessity of human intervention. It has shown promising results in detecting malignancies, radiological images, and in precision medicine for developing personalised treatments [15–17]. These models can learn from clinical data, data extracted from medical images (radiomics), or directly from medical imaging, which may support evidence-based decision-making in clinical practice. In CRLM management, these AI algorithms may enhance the accuracy of imaging interpretation, help optimise local treatment strategies, provide prediction tools to gain insight into treatment outcomes, and support in clinical decision-making at the individual patient level. Consequently, it may help overcome current limitations in CRLM management and result in more personalised and effective treatment strategies. Nonetheless, the clinical benefits have not yet been clearly demonstrated and are not yet validated in patients with CRLM.

The aim of this review is to assess the status of the use of AI models, particularly those based on ML, deep learning, and radiomics in surgical or TA treatment for patients with CRLM. This review will provide insight into the potential benefits and challenges associated with the integration of AI into the management of CRLM.

## Materials and methods

### Search strategy

This review was conducted in compliance with the Preferred Reporting Items for Systematic Reviews and Meta-Analyses Extension for Scoping Reviews (PRISMA-ScR) guidelines (appendix 1) [18]. A comprehensive literature search was conducted on the 15th of October 2024 (A.V.) across PubMed, EMBASE, Web of Science, and SCOPUS. The search strategy involved terms related to the research question. The following Medical Subject Headings (MeSH) terms and keywords were assessed: artificial intelligence, machine learning, colorectal liver metastasis, and liver metastases of the colon, local treatment, surgery and liver ablation. The final search algorithms are provided in Appendix 2.

### Study selection

Eligible studies were those including (1) patients diagnosed with CRLM and (2) undergoing liver surgery or TA, (3) AI for building prediction models, and (4) AI used for clinical decision-making. Only cohort studies and clinical trials were considered. Regarding AI, articles were included if they reported on ML techniques (e.g., support vector machine (SVM), random forests) and, if applicable, deep learning techniques (e.g., convolutional neural networks (CNNs) and transformers). Case reports, (systematic) reviews, conference abstracts, letters to the editor, and articles written in languages other than English were excluded. Studies where participants received systemic therapy were also excluded. The search results of all databases were imported in Endnote v21.1. After removal of duplicates, the articles were imported via Endnote into Rayyan. Two reviewers (A.V. and R.K.) screened the articles in a double-blind fashion. The reference lists of included studies were also screened to identify eligible articles. In case of disagreement, consensus was reached by discussion or, if necessary, by consulting a third reviewer (C.L.).

### Data extraction

Data were extracted using a predefined data extraction form by A.V. on the following variables: study population, treatment strategy, and type of model used for outcome (clinical, radiomics or combined). Clinical or radiomics parameters used in model (if applicable), and measurements used for primary outcome were extracted. Included articles were categorised based on the local treatment strategy. Regarding AI, the used techniques or models were extracted from the articles. The techniques were divided into: registration, segmentation, classification or survival/regression. Clinical outcomes were defined as treatment response, local tumour recurrence, recurrence of disease, and survival. For predictive clinical, radiomics or combined models, the following outcomes were extracted: area under the curve (AUC), sensitivity, specificity, positive predictive value, negative predictive value, accuracy of the model, concordance (C)–index, survival outcomes, and for radiomics, the imaging modality and features.

## Results

### Study selection

**Fig. 1:**
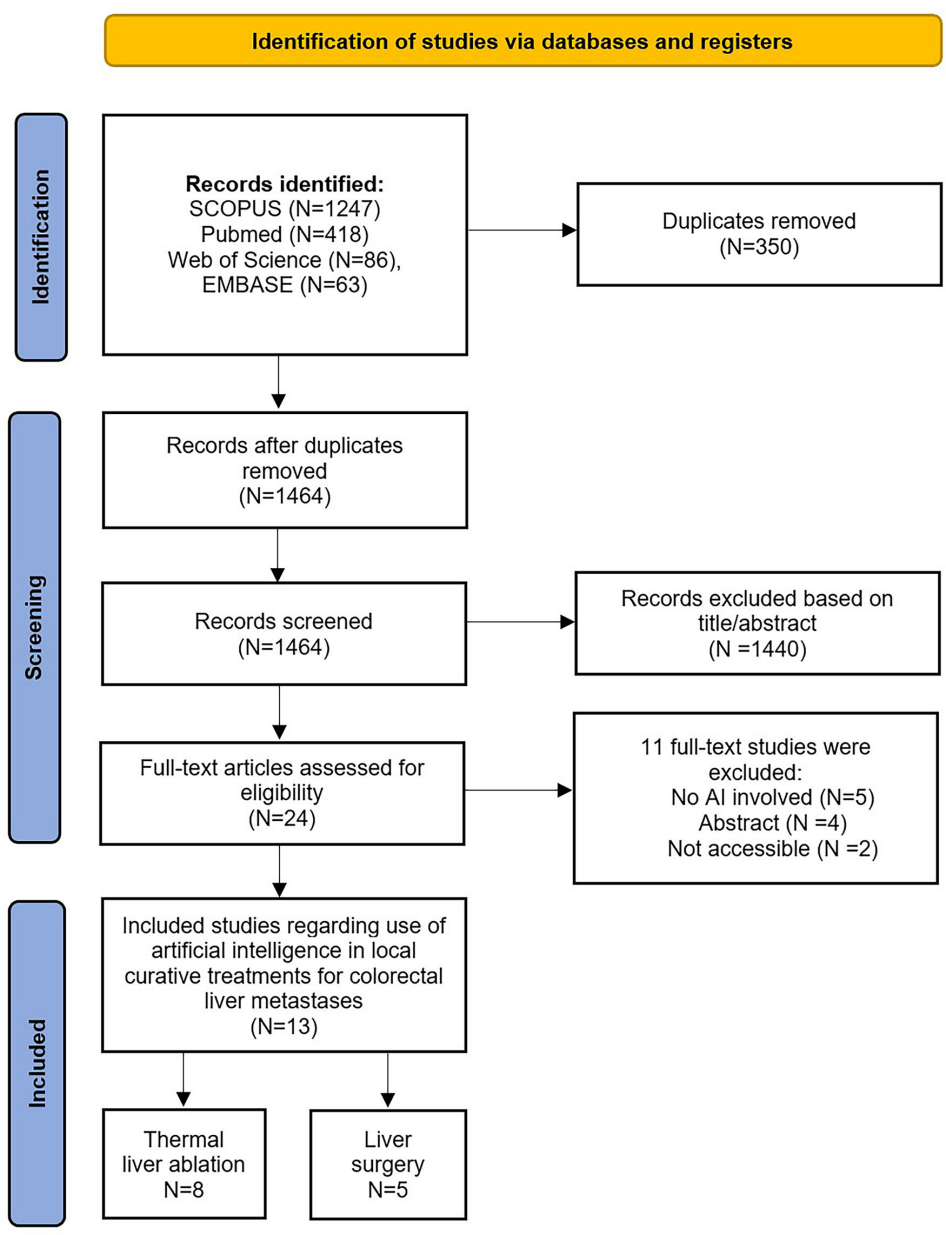
Flow diagram of the study selection

The search strategy yielded *n* = 1464 articles after removal of duplicates. A total of *n* = 24 articles underwent full-text screening, after 1440 articles were excluded based on title or abstract. Additionally, *n* = 11 articles were excluded due to insufficient reporting on the AI models used (*n* = 5), abstract only (*n* = 4) and inaccessibility to the article (*n* = 2). Eventually, *n* = 13 articles were included in this review (Fig. 1), *n* = 8 focused on TA and *n* = 5 on surgery [19–31]. Table 1 shows detailed information on included articles, AI methods used, and the outcome measurements described.

**Table 1:** Articles included in the review

Study	Year	Study design	Treatment	No of patients (tumours)			AI category	Clinical features	Clinical outcomes	Imaging		Imaging features	Outcome parameters
				Patients (lesions)	Training model	Validation model				Modality	Timing		
Anderson [19]	2022	R, single-centre	Thermal ablation (unspecified)	92	72	20	Segmentation	N/A	Disease site diameter	CT	Pre- and post-treatment	Mean surface distance	DSC, Mean Surface distance, sensitivity
Lin [20]	2023	R, single-centre	RFA/MWA	124 (213)	124	N/A	Registration/segmentation	Age, gender, Extrahepatic metastasis, chemotherapy, CEA, RAS, tumour characteristics	Local disease progression, MAM	CT	Pre- and post-treatment	DIR and autosegmentation for MAM	Cumulative incidence, SHR
Lin [21]	2024	R, single-centre	RFA/MWA	72 (139)	N/A	N/A	Registration/segmentation	Tumour characteristics	MAM, residual tumour, LTP at 1- and 2-year	CT	Peri-procedural	DIR and RIR for MAM	AUC-ROC, SHR
Paolucci [22]	2024	R, multicentre	RFA/MWA	243 (400)	N/A	N/A	Registration/segmentation	N/A	3-year local disease progression, overall survival	CT, ultrasound	Peri-procedural	Biomechanical DIR and autosegmentation for MAM	AUC-ROC, HR
Hu [23]	2023	R, single-centre	MWA following hepatic resection	318 (455)	216 (315)	102 (140)	Survival/regression	Age, gender, TNM-stage, initial tumour location, RAS, ablative margin, clinical risk score, time between resection and MWA, adjuvant therapy post MWA, CEA, CA19-9, no of target liver metastases, tumour characteristics	LTP, 3-year survival, 5-year survival	CT	Prior to ablation, after hepatic resection	RAD-score, GLCM, GLDM, GLZSM, GLRLM, FIRST ORDER, two-dimensional features for shape & size	AUC, sensitivity, specificity, PPV, NPV, accuracy, C-index
Shahveranova [24]	2022	R, single-centre	MWA	42 (67)	42 (67)	N/A	Survival/regression	Age, extrahepatic disease, KRAS, tumour size, INR, CA-19-9, CEA	LTP	MRI	Pre-treatment	GLCM, GLRLM, GLSZM, NGTDM, GLDM, FIRST ORDER, Shape	AUC, sensitivity, specificity, NRI, IDI
Della corte [25]	2024	R, single-centre study	MWA	43 (76)	N/A	N/A	Survival/regression	TNM staging, RAS mutational status, localisation in lower gastrointestinal tract, clinical risk score, history of previous therapies (systemic/local), tumour characteristics	LTP-free survival	MRI	Pre-treatment	morphology, Statistical, IG, GLRL3D_average, GLRL3D_comb, GLCM3D_average, GLCM3D_combined, GLSZM3D, NGTDM3D, GLDZM3D	C-index, sensitivity, specificity, HR
Taghavi [26]	2021	R, single-centre	RFA/MWA	94/82*	65/59*	29/23*	Survival/regression	Age, sex, tumour stage, primary ablation modality, ablation combined with surgery, (neo)adjuvant chemotherapy, CEA, tumour characteristics	Development of new CRLM	CT	Pre-treatment	GLCM, GLRLM, GLSZM, NGTDM, GLDM, FIRST ORDER	AUC
Granata [27]	2022	R, single-centre	Surgical resection	81 (151)	51 (121)	30 (30)	Survival/regression	N/A	Font of tumour growth, tumour budding, mucinous type, recurrence	MRI	Pre-treatment	GLCM, GLRLM, GLSZM, NGTDM, GLDM, FIRST ORDER	AUC, sensitivity, specificity, PPV, NPV, accuracy
Granata [28]	2022	R, single-centre	Surgical resection	81 (151)	51 (121)	30 (30)	Survival/regression	N/A	Font of tumour growth, tumour budding, mucinous type, recurrence	MRI	Pre-treatment	GLCM, GLRLM, GLSZM, NGTDM, GLDM, FIRST ORDER	AUC, sensitivity, specificity, PPV, NPV, accuracy
Granata [29]	2022	R, single-centre	Surgical resection	81 (151)	51 (121)	30 (30)	Survival/regression	N/A	Font of tumour growth, tumour budding, mucinous type, recurrence	MRI	Pre-treatment	GLCM, GLRLM, GLSZM, NGTDM, GLDM, FIRST ORDER	AUC, sensitivity, specificity, PPV, NPV, accuracy
Paredes [30]	2020	R, Multicentre	Surgical resection	1406	703	703	Survival/regression	Age, sex, primary tumour characteristics, no. of metastases in the liver, lymph node metastases, tumour size, CEA, KRAS mutation, chemotherapy before surgery	Recurrence-free survival	N/A	N/A	N/A	AUC, HR
Montagnon [31]	2024	R, single-centre	Systemic chemotherapy and surgery	241 (731)	85%	15%	Classification, survival/regression	Node positive primary cancer, disease-free interval from primary to CRLM diagnosis, CEA, hepatic tumour size	Time to recurrence, disease-specific survival	CT	Pre-treatment	GLCM, GLRLM, GLSZM, NGTDM, GLDM	C-index, AUC-ROC

*AUC* area under the curve, *CA-19-9* carbohydrate antigen, *CEA* carcinoembryonic antigen, *C-index* concordance index, *CRLM* colorectal liver metastasis, *cT* clinical tumour stage, *CT* computed tomography, *DSC* dice similarity coefficient, *DIR* deformable image registration, *GLCM* grey level co-occurrence matrix, *GLDM* grey level dependence matrix, *GLRLM* grey level run length matrix, *GLSZM* grey level size zone matrix, *IBS* integrated brier score, *IDI* integrated discrimination improvement, *INR* international normalised ratio, *KRAS* kirsten rat sarcoma virus, *LTP* local tumour progression, *MAM* minimal ablation margin, *MRI* magnetic resonance imaging, *MWA* microwave ablation, *NGTDM* neighbouring grey tone difference matrix, *NPV* negative predictive value, *NRI* net reclassification index, *PPV* positive predictive value, *R* retrospective, *RAD-score* radiomics score, *RAS* rat sarcoma, *RFA* radiofrequency ablation, *RIR* rigid image registration, *SHR* subdistribution hazard ratio, *TNM* tumour nodule metastasis

* Two training and validation cohorts were divided in the study of Taghavi et al: one for the prediction of colorectal liver metastases 6 months after ablation, and one for 24 months after ablation

### Thermal liver ablation

AI techniques in patients undergoing TA were used to develop predictive models for LTP, new CRLMs, and ablation zone segmentation on imaging. Seven included studies were conducted retrospectively in a single-centre setting [19–21, 23–26]. One study was a multicentre retrospective cohort study [22].

Four studies from MD Anderson Cancer Center focused on autosegmentation for ablation zone quantification in patients with CRLM using CT images [19–22]. Anderson et al investigated four fully CNN-based models for the identification and segmentation of CRLM and ablation zones [19]. The models were trained on 92 patients, with 72 patients included in the training model cohort and 20 patients in the validation cohort from the liver tumour segmentation (LiTS) challenge [32]. The evaluation cohort consisted of 15 patients from the 3D-IRCADb dataset [33], 18 patients from the institution, and 70 patients from the LiTS challenge [32]. The best trained model was the hybrid W-net, which demonstrated good performance for both CRLM target disease site (mean Dice similarity coefficient (DSC) = 0.80) and target ablation zones (mean DSC = 0.75). The model performance was better for lesions with a diameter ≥ 15 mm compared to lesions with a diameter < 15 mm (mean DSC = 0.74 versus 0.16, sensitivity: 98% versus 23%, respectively). Lin et al studied the use of AI-based deformable image registration (DIR) combined with autosegmentation on CT imaging for ablative margin quantification and its association with disease progression following TA at 1 and 2 years [20]. A total of 124 patients (*n* = 69 female; mean age = 57 (SD ± 12) years) treated with RFA or MWA with 213 CRLMs were included. Ablation margins ≥ 5 mm were achieved in 45% of the entire study population. An ablation margin of 0 mm was identified as a significant independent predictor of local disease progression, showing a subdistribution hazard ratio of 23.3 (95% CI: 10.8–50.5, *p* < 0.001) by the use of DIR and autosegmentation combined. Both DIR and autosegmentation enabled identification of CLRMs at risk for local disease progression, with an optimal minimal ablation margin (MAM) of 5 mm. Another study performed by Lin et al explored the comparison of DIR and rigid image registration (RIR) on CT imaging for MAM quantification after MWA or RFA, and its influence on LTP at 1 and 2-year post-TA [21]. A total of 72 patients (*n* = 28 female; mean age = 57 (SD ± 12) years) with 139 CRLMs were included. The study demonstrated no difference between DIR and RIR for MAM assessment (4.7 mm versus 4.0 mm; *p* = 0.08, respectively). For predicting residual tumour tissue and 1-year LTP, DIR demonstrated better performance compared to RIR with an AUC under the receiver operating curve (AUC-ROC) of 0.89 (95% CI: 0.83–0.94) versus 0.72 (95% CI: 0.61–0.83) (*p* < 0.001), respectively. Similar to the previous study [20], a MAM of 0 mm was quantified as a significant independent predictor of LTP, with a subdistribution hazard ratio of 9.3 (95% CI: 4.1–20.8, *p* < 0.001). A follow-up retrospective multicentre study was performed by Paolucci et al, to validate the previously identified MAM of 5 mm using the developed DIR combined with autosegmentation by Lin et al [20] for patients undergoing RFA or MWA for CRLM [22]. A total of 243 patients (*n* = 98 female; median age = 62 (range: 54–70) years) with 400 ablated CRLMs were included. A MAM ≥ 5 mm was achieved in 33%, and local disease progression within 3 years was observed in 20% across all institutions. The AUC-ROC of predicting local disease progression by MAM within 3 years for intraprocedural and peri-procedural imaging were 0.92 (95% CI: 0.86–0.98) and 0.77 (95% CI: 0.69–0.85), respectively, demonstrating that MAM ≥ 5 mm confirmed by DIR and autosegmentation have good oncological outcomes.

Hu et al developed a radiomics nomogram including clinical information to predict LTP at 3 years after MWA for patients diagnosed with recurrent CRLM following hepatic resection in a retrospective single-centre setting [23]. Radiomics features were extracted from CT images collected 1 month prior to TA. ML was used for the design of a clinical model, a radiomics model, and a combined model integrating clinical and radiomics features. SVM was used for model classification, and Bayesian hyperparameter optimisation was utilised for model performance. Clinical features are overviewed in Table 1. The cohort was divided into a training cohort consisting of 216 patients (*n* = 74 female; mean age = 62 (SD ± 11) years) with 315 lesions, and a validation cohort consisting of 102 patients (*n* = 35 female; mean age = 61 (SD ± 10) years) with 140 lesions. The radiomics-based nomogram demonstrated high predictive performance in the training cohort (ACC = 96%; AUC = 0.95) and in the validation cohort (ACC = 85; AUC = 0.89), compared to the radiomics model and clinical model separately.

Shahveranova et al developed a combined model based on clinical features and MRI radiomics features with an ML approach (unspecified) to predict LTP after MWA in a retrospective single-centre design at 3 or 6 months follow-up [24]. The population consisted of 42 patients (*n* = 16 female; mean age = 55.4 (SD ± 10.0) years) with 67 lesions. A clinical model, a radiomics model and two combined models, one consisting of T1 fat-suppressed sequences (phase 1) and one of T2 fat-suppressed sequences (phase 2), were developed. The clinical model consisted of extrahepatic disease status, tumour size, and CA19-9. The phase-2 combined model had the highest discriminative performance in predicting LTP (AUC = 0.98; 95% CI: 0.95–0.99) compared to the other models. No, external validation was performed.

Another study exploring the predictive value of pre-procedural MRI radiomics features was performed by Della Corte et al on LTP-free survival after MWA for patients with CRLM [25]. An ML bootstrap-based ranking method was used to develop predictive models. Internal validation criteria of the TRIPOD guidelines were followed to test the models [34]. A total of 43 patients (*n* = 18 women; mean age = 69 years) with 76 CRLMs were included. The following predictive models were developed: a clinical model (CLIN-1) consisting of MAM, intra-segment progression and tumour grade, a radiomics model consisting of radiomics features from T2 (RAD-T2) and T1-hepatobiliairy phase (RAD-T1) sequences, and two combined models (COMB-T1 and COMB-T2) according to the T1-hepatobiliairy phase and T2 sequences, respectively. All four models effectively predicted LTP-free survival, with COMB-T1 (AUC = 0.98, *p*-value < 0.001) and COMB-T2 (AUC = 0.95, *p*-value < 0.001) reaching the highest predictive value. No external validation was performed for the models.

Taghavi et al investigated whether radiomics features from pre-ablation CT images could predict new CRLMs after TA in a retrospective single-centre setting [26]. The study population was divided into a training cohort (65 patients; *n* = 29 female; mean age = 60 (SD ± 10) years) and a validation cohort (29 patients; *n* = 8 female; mean age = 63 (SD ± 11) years). First, an original model with radiomics developed to predict new CRLMS 6 months post-ablation, published previously by the research group, was utilised [35]. Next, three new models were developed with ML: a clinical, radiomics, and combined (clinical and radiomics) model. The classification model was built using random forest, and Bayesian hyperparameter optimisation was used for tuning hyperparameters in the training set. The ‘original’ model showed low predictive performance with an AUC of 0.57 (95% CI: 0.56–0.58) for 6 months, and for 24 months (AUC = 0.52; 95% CI: 0.51–0.53). The newly developed models also showed low accuracy for predicting new CRLMs. At 6 months, the combined model showed a slightly better performance (AUC = 0.60; 95% CI: 0.59–0.61) compared to the radiomics (AUC = 0.57; 95% CI: 0.56–0.58) and clinical model (AUC = 0.55; 95% CI: 0.53–0.54) in the training sets. For 24 months, the combined model and radiomics model showed both good accuracy (AUC = 0.76; 95% CI: 0.75–77) for the training set. However, the original and new models both resulted in low accuracy (AUC = 0.53–57) for both follow-up moments when used for the validation set.

Six studies demonstrated that AI-driven models have high performance in predicting treatment outcomes. Four studies conducted at the MD Anderson Cancer Center (United States of America) focused on autosegmentation and DIR of the ablation zone and showed good performance for targeting disease site and ablation margin confirmation developed with AI models [19–22]. This was also confirmed in a multicentre retrospective centre, showing optimal oncological outcomes for MAMs of 5 mm. For LTP, combined models show better performance for LTP than clinical or radiomics models separately. High accuracy (AUC: 0.95–0.98) was observed by Della Corte et al, Hu et al and Shahveranova et al [23–25]. The models of Taghavi et al showed low predictive performance when validated, which may suggest that ML models may be population specific [26].

### Surgical resection

In five retrospective studies regarding surgery for CRLM, AI was utilised for predictive modelling with clinical data and/or radiomics features [27–31].

Three retrospective studies were conducted by Granada et al in 2022, investigating the use of pre-surgical MRI-based radiomics with ML approaches to predict clinical outcomes (recurrence, front of tumour (expansive versus infiltrative growth), mucinous type, and tumour budding (high grade versus low grade, or absent)) [27–29]. Pattern recognition techniques using ML algorithms such as SVM, k-nearest neighbours (KNN), artificial neural network (NNET), and decision tree were used for model building. In all three studies, the same cohort consisting of 81 patients (*n* = 28 female; median age = 61 (range: 35–82) years) with 151 lesions was included. In the training model, 51 patients (*n* = 18 female; median age = 61 (range: 35–82) years) with 121 lesions were included. The external validation cohort consisted of 30 patients (*n* = 10 female; median age = 60 (range: 40–78) years) with 30 CRLMs. The authors used the same 851 radiomics features. In one preliminary study, traditional ML and deep learning structures were used to predict the four clinical outcomes for patients following liver resection on pre-surgical multiphase MRI [27]. The radiomics models demonstrated high performance, especially on arterial phase (accuracy: 0.89–0.94) compared to portal venous phase (accuracy: 0.83–0.92), considering extracted radiomics features for the clinical outcomes. The best performance of each outcome was reached by a KNN classifier. The same research team also assessed the efficacy of radiomics features obtained by pre-surgical T2-weighted MRI sequences [28]. The best performing models showed an accuracy ranging from 82% to 92% for all four above-mentioned clinical outcomes. With regard to ML approaches, the best performance in discrimination for tumour growth was reached by a decision tree, while discrimination of tumour budding, mucinous type, and presence of recurrence was best reached with KNN. Additionally, the utility of gadoxetic acid-enhanced (EOB) MRI-based radiomics features in prediction models for clinical outcomes was also investigated [29]. The best radiomics feature accuracies ranged from 81% to 89%. Linear regression obtained good results in each clinical outcome with accuracies ranging from 72% to 89%. The best performance for each clinical outcome with pattern recognition approaches was reached by a KNN, with an accuracy ≥ 80%.

In 2020, Paredes et al published a retrospective multicentre study with the aim of developing a recurrence prediction model with clinical features for patients undergoing curative hepatic resection for CRLM [30]. The model design cohort consisted of 703 patients (*n* = 286 female; median age = 60.5 (range: 52.0–68.0) years), and the validation cohort consisted also of 703 patients (*n* = 271 female; median age = 61 (range: 53.1–68.0) years). The final developed prediction models were based on clinical features overviewed in Table 1. In one model, known Kirsten RAS (KRAS) status was included. Predictive model building was performed using bootstrap resampling methodology in combination with multivariable mixed-effects logistic regression analysis. The model without KRAS status showed the ability to predict the risk of recurrence at 1-, 3- and 5-year follow-up for the validation cohort with AUC-ROC of 0.65 (95% CI: 0.64–0.66), 0.66 (95% CI: 0.65–0.67) and 0.66 (95% CI: 0.65–0.67), respectively. The alternative model for the validation cohort with KRAS status showed the ability to predict risk of recurrence at 1-, 3- and 5-year follow-up with AUC-ROC: 0.64 (95% CI: 0.64–0.63), 0.66 (95% CI: 0.66–0.67) and 0.67 (95% CI: 0.66–0.67), respectively. Both models were compared to the existing CRS of Fong-Blumgart [36] and Vauthey [37], and showed more accurate predictive performance than the two existing scores.

Montagnon et al analysed a prospectively maintained registry of patients treated with systemic chemotherapy and curative surgery for CLRM to compare the performance of pre-treatment CRS, radiomics models based on CT imaging, and a combined model of radiomics and CRS for predicting time to recurrence and disease-specific survival [31]. CRS was calculated using preoperative data similar to the CRS of Fong-Blumgart [36]. Radiomics features were extracted on pre-treatment CT scans of 731 manually segmented CRLMs. ML algorithms such as random survival forest from the Scikit-Survival [38] package and neural network survival models from DeepSurv [39] were utilised for the development of models. The study population consisted of 241 patients (*n* = 86 female; mean age = 62.9 (SD ± 9.5) years). The radiomics-based models, including CRS outperformed the traditional CRS for time to recurrence showing a C-index of 0.70 (95% CI: 0.68–0.73) for DeepSurv and for random survival forest 0.61 (95% CI: 0.60–0.61), compared to a C-index of 0.57 (95% CI: 0.57–0.57) for CRS alone. In disease-specific survival prediction, the C-indexes were 0.59 (95% CI: 0.59–0.59), 0.60 (95% CI: 0.58–0.61), 0.57 (95% CI: 0.56–0.57), for CRS, DeepSurv combined with CRS, and random survival forest combined with CRS, respectively. No validation was performed.

These studies demonstrated that clinical models and CT- and MRI-based radiomics models developed by ML techniques have the potential to predict recurrence after surgical resection for CRLM. Performance of radiomics-based models shows better performance than clinical models alone. However, only one study used clinical characteristics for model development [30]. Moreover, the added value of these models in clinical practice and the aid of AI/ML in the decision-making process of therapeutic choices is not clearly stated.

## Discussion

This review summarises the findings of studies that used AI technologies in the treatment pathway of patients with CRLM. Different AI-based models for the development of prediction models for TA and surgical resection for CRLM were discussed. These models utilised clinical information, imaging data, including CT and MRI, and genetic data. Ten studies demonstrated that predictive models developed with AI showed high performance for clinical outcomes in patients with CRLM [19, 20, 23–25, 27–31]. More importantly, the predictive value improved when a combined model was developed for recurrence, tumour progression, or survival.

The AI methods used in the articles, with the exception of Anderson et al [19], predominantly used traditional machine learning (ML) techniques such as SVM, KNN, NNET, random forests, and decision trees. These techniques are often combined with traditional radiomics, which uses predefined “hand-crafted” features that attempt to describe medical images. A limitation of this approach is that each individual radiomics feature may contain very little signal and predictive value. This requires extensive stability testing to ensure that the identified “winning” feature remains top-ranking even after thousands of bootstrap resampling iterations. Unfortunately, none of the studies performed stability or robustness tests, and the reported feature *p*-values lack this essential validation. Moreover, the sample sizes are often relatively small compared to the number of features analysed, which may result in misleading and overoptimistic outcomes. Therefore, any conclusions drawn from these studies should be approached with caution. In contrast, more recent deep learning-based AI models, as discussed by Anderson et al [19], have a well-established ability to provide comprehensive and predictive analysis of medical images. Their mechanism is, however, often a so-called “black box,” which can be a problem for clinical interpretation and external validation, as are high-level radiomics features, that may be equally incomprehensible to most clinicians and may also lack stability. Recent efforts to make deep learning-based AI methods more explainable are helping to address this “black box” nature, but there remains substantial room for improvement in applying modern AI methods for predictive purposes in CRLM diagnosis and treatment.

The use of radiomics to create prognostic models has gained interest in recent years [40–42]. A significant advantage of radiomics is its ability to analyse tumour features at a subregional level, finding features that cannot be assessed by the human eye. This approach may contribute to early detection of newly developed CRLM, local recurrence, or insufficient ablation margins. AI-driven approaches show promising results in the extraction and analysis of features retrieved from CT and MRI images [21–29, 31]. However, these studies showed variability in the imaging modality used for predictive modelling, the time point of feature extraction in the timeline of treatment planning, the methodology of feature extraction, and predictive model development with the use of AI strategies. Moreover, external validation often lacks in the performed studies, which severely limits the generalisability of the developed prediction models and clinical acceptance of these models. A standardised workflow in the extraction of radiomics features is lacking, which may result in variability in the performance of AI-driven predictive models in different clinical settings. Moreover, the radiomics features extracted may differ between CT and MRI, since sensitivity in CRLM detection and lesion characterisation differ between both modalities [43]. Thus, standardised methods of data collection and reporting guidelines are necessary for ensuring reproducibility and applicability in clinical settings. To illustrate, many studies do not conform to reporting guidelines such as TRIPOD, CLAIM and DECIDE [34, 44, 45]. The TRIPOD guidelines were only used in the study of Paredes et al and Della Corte et al [25, 30, 34]. Additionally, given the small sample sizes and limited number of institutions participating often seen in these studies, there is a risk of bias in the models. Consequently, the PROBAST tool may be considered in future studies for risk estimation [46, 47].

AI support tools show promising results in clinical practice, but some challenges need to be addressed. As mentioned previously, the ”black box” nature may impede the implementation of AI into clinical practice as clinicians may not understand the decisions or predictions made by AI algorithms [48, 49]. The interpretability of these models is important so that the AI support system can be understood. To overcome this challenge, a multidisciplinary team consisting of experts of different fields (e.g., (bio) medical, legal, computer science, socio-economics and patient advocacy) should collaborate to develop and validate AI algorithms. This would help with the identification of current barriers in CRLM workflow, and alignment of AI tools with clinical needs, eventually enabling a personalised medicine approach [50]. By integrating clinical and radiological data, AI algorithms may predict patient-specific recurrence risk, facilitate (real-time) ablation zones, and ultimately reduce the number of re-ablations or ineffective treatments. Among several prognostic factors, ablation margin is the most critical factor influencing recurrence risk, as insufficient ablation margin has been shown to be an independent risk factor of LTP [20, 21, 51–53]. This was also demonstrated in the studies of MD Anderson, where intraprocedural, real-time ablation margin quantification by AI-based autosegmentation and biomechanical DIR allowed precise 3D assessment of the MAM [19–22]. To predict recurrence risk more accurately and tailor patient care, it is also essential to incorporate additional tumour-specific features, including histopathological and imaging characteristics. An optimal predictive framework should synthesise multimodal data, encompassing procedure-related parameters (e.g., ablation margin), as well as patient- and lesion-specific features. This comprehensive integration would facilitate precise, individualised prognostic and therapeutic guidance. Such enhancements in AI-based tools hold promise for improving clinical outcomes while also increasing cost-effectiveness. As AI is a rapidly evolving field, having up-to-date knowledge and facilities to remain aligned with emerging technologies, existing ethical norms, and clinical practices is important. Given the pace of innovation, current evidence and methodology may quickly become incomplete or outdated. Although existing evidence will not become obsolete, the dynamic nature of AI innovation and potential improvement in model performance emphasise the importance of continuous evaluation. Second, the studies included used different outcome parameters, AI techniques and validation methods for the predictability of one treatment. Currently, predictive models are developed to assess clinical outcomes, such as survival and complications of patients diagnosed with CLRM, rather than to guide patient selection or inform treatment strategy. For patients with hepatocellular carcinoma, an ML-based clinical decision model has been developed by Choi et al [54]. In this retrospective study, the clinical decision support system, developed for different treatment strategies for 1021 patients with hepatocellular carcinoma, showed good performance with an accuracy of 81.0% for RFA or resection versus non-curative treatments, and 88.4% for RFA versus resection and 76.8% for transarterial chemoembolisation versus alternative treatment options. However, in a recent multicentre study in which this model was externally validated, the accuracy in the external dataset for the first treatment option dropped to 55.3% [55]. The accuracy improved to 86.1% in the external dataset when a second treatment option was included, and was considered the correct answer. The authors attributed the low accuracy of only the first treatment option to the heterogeneity of treatment options in different centres and the retrospective nature of the two datasets. Another study of Han et al developed an ML algorithm that facilitated treatment selection for the advanced stage of hepatocellular carcinoma and could predict survival for 6, 12 and 24 months, showing AUC-ROC ≥ 0.80 [56]. These studies suggest that AI can play a vital role in support tools for clinical decision-making and patient selection; however, both studies did not include radiomics features in their models. Since hepatocellular carcinoma differs histopathologically from CRLM and patient profiles also vary, these models cannot be directly translated to patients with CRLM.

To our knowledge, no AI-based clinical decision models have been developed yet for patients with CRLM. To address this issue, large (international) multicentre studies with multicentre validation cohorts are necessary, as conducted by Paolucci et al and Paredes et al [22, 30]. The ongoing large international, multicentre cohort study/registry on TA titled Ablation-IMaging and Advanced Guidance for workflow optimisation in Interventional Oncology (A-IMAGIO) is anticipated to contribute to the field of knowledge (clinicaltrials.gov ID: NCT06179602). In this study, clinical and radiomics data will be collected from patients with hepatocellular carcinoma, CRLM and other secondary liver tumours. AI-guided computational models will be developed to help guide decision-making for personalised treatment, optimise treatment planning, and identify patients at risk of recurrence.

This review has some limitations. First, meta-analyses or statistical analyses could not be performed due to the heterogeneity of the outcomes discussed in the included articles regarding statistical analyses, methodologies for model building, different time points of LTP or recurrence, and the objectives of the studies. Second, most studies utilised radiomics features for developing predictive models, with little to no consideration for other data sources such as genomic data, clinical decision support systems, and treatment planning with the help of AI. This resulted in an imbalance in the modelling techniques that were analysed, limiting the comprehensiveness of the review. Third, most included studies had small sample sizes in the test and validation cohorts, which limited the generalisability of the findings and hindered the identification of consistent modelling patterns and/or feature identification that are associated with improved prognostic performance.

Many AI algorithms are designed for specific patient populations because of the small sample sizes and single-centre study design used, limiting their clinical adaptation. For AI implementation in CRLM care, (international) multicentre studies, with prospective and standardised data collection of both clinical and imaging features, are necessary. Long-term follow-up data should also be included to train and evaluate AI algorithms on recurrence rates and patient outcomes. Additionally, external validation is necessary to ensure generalisability. This approach reduces selection bias, considers population heterogeneity, and will result in robust models that can be widely applied. Moreover, using AI reporting guidelines further minimises bias and enhances the reproducibility of AI models, enabling their integration into clinical practice. For future research, it is necessary to gain more insight into the association of clinical characteristics, histopathological tumour characteristics, and imaging features, such as (real-time) ablation zone segmentation, including margin quantification for predictive model development. Moreover, cross-institutional prospective cohort studies with large sample sizes and longitudinal patient follow-up data are necessary to develop and validate robust AI algorithms. Standardised work protocols and multidisciplinary expert teams for AI research should be implemented to limit heterogeneity in study outcomes and improve the interpretability of AI models that will be used in CRLM care.

## Conclusion

AI techniques show promising results in the development of prediction models for clinical outcomes of TA and surgical resection for CRLM. However, deep learning models have not yet been applied in patient selection, clinical decision support systems, and treatment planning for CRLM, and prospective data collection is lacking. Multicentre studies with large training datasets, external validation cohorts, and standardised workflows are necessary to facilitate personalised and effective treatment strategies to eventually enhance clinical outcomes for patients with CRLM with the support of AI tools.

## Supplementary information

ELECTRONIC SUPPLEMENTARY MATERIAL

## Data Availability

The data analysed during this study are included in this published article.

## Abbreviations

AI: Artificial intelligence
AUC: Area under the curve
AUC-ROC: Area under the receiver operating curve
CA 19-9: Carbohydrate antigen
CEA: Carcinoembryonic antigen
C-index: Concordance-index
CNN: Convolutional neural networks
CRLM: Colorectal liver metastases
CRS: Clinical risk score
DIR: Deformable image registration
DSC: Dice similarity coefficient
KNN: k-Nearest neighbours
KRAS: Kirsten rat sarcoma virus
LiTS: Liver tumour segmentation
LTP: Local tumour progression
MAM: Minimal ablation margin
ML: Machine learning
MWA: Microwave ablation
NNET: Artificial neural network
PRISMA-ScR: Preferred Reporting Items for Systematic Reviews and Meta-Analyses Extension for Scoping Reviews
RAS: Rat sarcoma virus
RFA: Radiofrequency ablation
RIR: Rigid image registration
SVM: Support vector machine
TA: Thermal ablation
TNM: Tumour, nodes and metastasis
